# Multiple colorectal adenomas in Lynch syndrome

**DOI:** 10.3389/fonc.2022.1038678

**Published:** 2022-12-20

**Authors:** Ayushi Jain, Maryam Alimirah, Heather Hampel, Rachel Pearlman, Jianing Ma, Jing Peng, Matthew F. Kalady, Peter P. Stanich

**Affiliations:** ^1^ Department of Internal Medicine, The Ohio State University Wexner Medical Center, Columbus, OH, United States; ^2^ Division of Gastroenterology, Hepatology and Nutrition, The Ohio State University Wexner Medical Center, Columbus, OH, United States; ^3^ Division of Clinical Cancer Genomics, City of Hope National Medical Center, Duarte, CA, United States; ^4^ Division of Human Genetics, The Ohio State University Wexner Medical Center, Columbus, OH, United States; ^5^ Center for Biostatistics, Department of Biomedical Informatics, The Ohio State University Wexner Medical Center, Columbus, OH, United States; ^6^ Division of Colorectal Surgery, The Ohio State University Wexner Medical Center, Columbus, OH, United States

**Keywords:** lynch syndrome, adenomas, colon cancer, multiple adenomas, Lynch

## Abstract

**Background:**

Lynch syndrome has not traditionally been considered to have a high colorectal adenoma burden. However, with increasing adenoma detection rates in the general population, the incidence of adenoma detection in Lynch syndrome may also be increasing and leading to higher cumulative adenoma counts.

**Aim:**

To clarify the prevalence and clinical impact of multiple colorectal adenomas (MCRA) in Lynch syndrome.

**Methods:**

A retrospective review of patients with Lynch syndrome at our institution was performed to assess for MCRA (defined as ≥10 cumulative adenomas).

**Results:**

There were 222 patients with Lynch syndrome among whom 14 (6.3%) met MCRA criteria. These patients had increased incidence of advanced neoplasia (OR 10, 95% CI: 2.7-66.7).

**Conclusions:**

MCRA is not unusual in Lynch syndrome and is associated with a significantly increased likelihood of advanced colon neoplasia. Consideration should be given to differentiating colonoscopy intervals based on the presence of polyposis in Lynch syndrome.

## Introduction

Inherited colorectal cancer syndromes are often categorized into “polyposis” and “nonpolyposis” syndromes (1). Lynch syndrome is a hereditary cancer syndrome characterized by heterozygous pathogenic variants in *MLH1*, *MSH2*, *MSH6*, or *PMS2* or an *EPCAM* deletion. Existing literature in Lynch syndrome initially suggested the majority of patients carry *MLH1* or *MSH2* pathogenic variants, although analysis of multigene panel testing results suggest that the frequency is likely similar across the genes and analysis of international registries predicts that *MSH6* and *PMS2* pathogenic variants may be more common (2, 3). Lynch syndrome has classically been associated with a lower colorectal adenoma burden and considered a nonpolyposis syndrome (4, 5). There is limited data on the adenoma burden in patients with Lynch syndrome, though one previous estimate suggested a mean of 7 adenomas by age 80 (6). Another study reported 4% of patients with Lynch syndrome had ≥ 10 cumulative lifetime adenomas and qualified as having a clinical oligopolyposis syndrome, contrary to the traditional assumption that patients with Lynch syndrome do not meet this criteria (7). In addition, with recent reports of nationwide increases in adenoma detection rates, the cumulative lifetime adenoma counts in the Lynch syndrome population are likely increasing along with the general population (8). As such, Lynch syndrome should also be considered in the differential of patients meeting multiple colorectal adenomas criteria, defined as 10 or more adenomas (9).

Lynch syndrome patients have long been recommended to have a colonoscopy every 1-2 years in the United States (1, 10). The latest guidelines from the Mallorca group are now recommending colonoscopy surveillance intervals of 2 to 3 years for most genotypes and up to 5 years for those with *PMS2* mutations (11). However, recent studies have shown that patients with ≥ 10 cumulative lifetime adenomas are at higher risk of advanced neoplasia and colorectal cancer (12). It is unclear if Lynch syndrome patients that meet this criterion would also have additional increased risks.

Our aim was to assess the prevalence of multiple colorectal adenomas in Lynch syndrome and assess for an association with advanced colorectal neoplasia and colorectal cancer.

## Methods

This was a retrospective study assessing patients with Lynch syndrome followed in the Hereditary and High-Risk Gastroenterology Clinic. Institutional Review Board approval was obtained prior to study initiation.

Inclusion criteria for the study were age 18 years or greater, a documented pathogenic or likely pathogenic variant in a mismatch repair gene (*MLH1, MSH2, MSH6, PMS2)* or in *EPCAM* on germline genetic testing, the completion of at least one colonoscopy at our institution and a clinic visit from August 2014 through December 2020. Exclusion criteria included a known pathogenic or likely pathogenic variant in additional hereditary cancer genes and a history of total colectomy prior to identification of first adenomatous polyp. All available colonoscopy and pathology records were reviewed to identify polyp characteristics. Colonoscopies were performed with the available endoscopic technology at the time of completion, which included both standard definition and high-definition white light endoscopy and virtual chromoendoscopy. Dye chromoendoscopy was not utilized.

The primary study outcome was the prevalence of multiple colorectal adenomas, defined as ≥ 10 lifetime tubular adenomas (9). The secondary outcomes of interest included prevalence of previous advanced colorectal neoplasia and colorectal cancer in those with and without MCRA. Advanced colorectal neoplasia was defined as a lifetime history of colorectal cancer, advanced adenoma, or advanced sessile serrated lesion. Advanced adenomas were defined as adenomas ≥ 10 mm in size, villous or tubulovillous adenomas or adenomas with high-grade dysplasia. Advanced sessile serrated lesions were defined as sessile serrated polyps ≥ 10 mm in size or with features of high-grade or low-grade dysplasia.

Statistical analysis included univariable analysis to compare patients with 0-9 tubular adenomas and ≥ 10 tubular adenomas. Odds ratio and 95% confidence interval was reported using univariable logistic regression for all variables. Multivariable analysis was utilized to adjust for age.

## Results

Two hundred and twenty-two patients met study criteria and were included in the analysis with demographic and clinical details available in **Table 1**. There were 142 patients (64%) with adenomas in the cohort and 14 patients (6.3%) met criteria for MCRA with 10 or more cumulative adenomatous polyps. The patients with MCRA had a mean of 7 colonoscopies available for review but the majority of patients with MCRA required 3 or less procedures to reach this criterion (13/14, 92.9%). The highest MCRA count was 28 adenomas. The MCRA patients had a mean age of 62 and the two most common mutated genes were *MSH6* (8/14) and *MSH2* (4/14). Of note, 12 (86%) had a history of advanced neoplasia including 5 (36%) with colorectal cancer.

**Table 1:** Characteristics of patients with Lynch syndrome.

Characteristic	N = 222
**Age** (Mean, SD)	48.03 (13.36)
**Sex**	
Female	151 (68%)
Male	71 (32%)
**Race**	
African American	3 (1.5%)
Asian	4 (2%)
Caucasian	212 (95%)
Hispanic	1 (0.5%)
Other	2 (1%)
**Gene**	
*MLH1*	40 (18%)
*MSH2*	58 (26%)
*MSH6*	75 (34%)
*PMS2*	49 (22%)
**BMI** (log)	3.35 (0.24)
**Daily Aspirin**	100 (45%)
**Colectomy**	
None	180 (81%)
Partial	39 (18%)
Total	2 (1%)
**Any Cancer**	102 (46%)
**Colorectal Cancer**	41 (18%)
**Family History of CRC**	164 (76%)
FDR with CRC	98 (46%)
SDR with CRC	128 (59%)
**Total Polyps**	
0 – 9	187 (84%)
≥ 10	35 (16%)
**Adenomas**	
0 – 9	208 (93.7%)
≥ 10	14 (6.3%)
**Sessile Serrated Adenoma**	
None	173 (78%)
≥ 1	49 (22%)
**Advanced Neoplasia**	89 (40%)

BMI, body mass index; CRC, colorectal cancer, FDR, first degree relative; SDR, second degree relative.

Univariable analysis was performed to compare the cohorts with 0-9 MCRA and ≥ 10 MCRA. Patients with ≥ 10 MCRA were older with a mean age of 62 as compared to 47 in patients with 0-9 MCRA (OR: 1.09, 95% CI: 1.04, 1.15), otherwise the cohorts had similar demographics including similar rates when compared within mismatch repair gene cohorts (**Table 2**). Patients with ≥ 10 MCRA were significantly more likely to have a history of advanced colorectal neoplasia as compared to patients with only 0-9 MCRA (OR: 10.2, CI: 2.69, 66.7). Notably, this remained true on multivariable analysis adjusted for age (OR: 5.35, CI: 1.34, 35.88). Patients with ≥ 10 MCRA were also more likely to have a personal history of malignancy (OR: 3.12, CI: 1.01, 11.7), although there was not a detected significant difference in a history of colorectal cancer.

**Table 2:** Univariable analysis of patients with adenomatous oligopolyposis.

Characteristic	0-9 AdenomasN = 208*1*	10+ AdenomasN = 14*1*	OR	95% CI
**Gene**				
*MLH1*	39/208 (19%)	1/14 (7.1%)	–	-
*MSH2*	54/208 (26%)	4/14 (29%)	2.89	0.41, 57.7
*MSH6*	67/208 (32%)	8/14 (57%)	4.66	0.81, 88.0
*PMS2*	48/208 (23%)	1/14 (7.1%)	0.81	0.03, 21.0
**Age**	47.11 (13.05)	61.71 (10.28)	**1.09**	**1.04, 1.15**
**Sex**				
Female	144/208 (69%)	7/14 (50%)	–	–
Male	64/208 (31%)	7/14 (50%)	2.25	0.74, 6.83
**BMI (log)**	3.35 (0.24)	3.35 (0.26)	1	0.09, 9.04
**Daily Aspirin**	96/208 (46%)	4/14 (29%)	0.46	0.49, 1.44.
**Race**				
Caucasian	200/208 (96%)	12/14 (86%)	-	-
Non-Caucasian	8/208 (3.8%)	2/14 (14%)	4.17	0.59, 19.0
**FDR with CRC**	90/199 (45%)	8/14 (57%)	1.61	0.54, 5.07
**Any Cancer**	92/207 (44%)	10/14 (71%)	**3.12**	**1.01, 11.7**
**Colorectal Cancer**	36/208 (17%)	5/14 (36%)	2.65	0.78, 8.17
**Advanced Neoplasia**	77/208 (37%)	12/14 (86%)	**10.2**	**2.69, 66.7**

1 n/N (%); Mean (SD)

OR, Odds Ratio; CI, Confidence Interval; FDR, first degree relative; CRC, colorectal cancer. Bold values indicate statistically significant values.

## Discussion

Lynch syndrome has traditionally been defined as a non-polyposis syndrome and differentiated from polyposis syndromes through a lower adenoma burden (4). In this study, we found that 6% of patients with Lynch syndrome had 10 or more cumulative adenomatous polyps and met MCRA criteria. This is similar to rates of MCRA seen in the general population in a recent study by Sullivan et al. (12). Although this is still a minority of patients with Lynch syndrome, these findings are notable as it is likely that the prevalence of colorectal adenomas in Lynch syndrome will continue to rise in conjunction with increases in adenoma detection rates in the general population. Given this, the mismatch repair genes should be included in multigene panel testing conducted for patients with MCRA as reflected in recent guidelines (13).

Our analysis also revealed that 86% of patients with Lynch syndrome and MCRA had a history of advanced neoplasia and that this was significantly higher than Lynch syndrome patients without polyposis even with adjustment for age. Similarly, Sullivan et al. found that patients without known inherited colorectal syndromes with ≥ 10 MCRA were 17 times more likely to have a history of advanced neoplasia (12). Together, these results indicate that MCRA is a high-risk phenotype for advanced neoplasia independent of genotype and should be taken into consideration to guide individualized recommendations for colorectal cancer surveillance.

A previous international cohort study presented evidence of the existence of unknown familial risk factors that result in wide variations in the risk of colorectal cancer across patients with Lynch syndrome (14). We propose that an MCRA phenotype may be a risk factor for advanced neoplasia and malignancy in patients with Lynch syndrome and should be given consideration when determining surveillance colonoscopy intervals. As guidelines are shifting toward longer colonoscopy intervals, we should consider that patients with a polyposis phenotype independent of Lynch syndrome genotype are likely high risk for advanced neoplasia and malignancy and may benefit from continued close monitoring.

Limitations of our study include the risk of ascertainment bias due to its use of genetic testing results as an inclusion criterion. Additionally, the majority of patients were Caucasian which may limit generalizability across diverse populations. It should also be noted that the majority of our patients carried *MSH6* and *PMS2* variants and thus may not be representative of all Lynch syndrome cohorts. In addition, the use of aspirin may have impacted advanced neoplasia in our patient population and could have a potential confounding effect. Large multi-center and prospective studies are needed to confirm the risks of polyposis in Lynch syndrome and to assess optimal colonoscopy intervals for these patients.

In summary, MCRA phenotype is not unusual in Lynch syndrome and was associated with a significant increase in history of advanced colon neoplasia. Given this, consideration should be given to individualizing colonoscopy intervals based on the presence of polyposis in Lynch syndrome.

## Data availability statement

The raw data supporting the conclusions of this article will be made available by the authors, without undue reservation.

## Ethics statement

The studies involving human participants were reviewed and approved by Ohio State Institutional Review Board. Written informed consent for participation was not required for this study in accordance with the national legislation and the institutional requirements.

## Author contributions

AJ and PS: conception and design, data collection, drafting of the article, critical revision of the article for important intellectual content, analysis of data, and final approval of the article. MA: data collection, analysis of the data, critical revision of the article for important intellectual content and final approval. JM and JP: Data analysis and final approval. HH, RP, and MK: critical revision of the article for important intellectual content and final approval. All authors contributed to the article and approved the submitted version.
